# Supplementary material for: Exploring the upper pH limits of nitrite oxidation: diversity, ecophysiology, and adaptive traits of haloalkalitolerant Nitrospira
Source: ISME J. 2020 Jul 24;14(12):2967–79. doi: 10.1038/s41396-020-0724-1 (PMC7784846; doi:10.1038/s41396-020-0724-1)
Supplement: Supplementary file 1 — Supplemental text [file 41396_2020_724_MOESM1_ESM.docx]

**Supplemental Text**

**Supplemental Materials and Methods**

Enrichment of alkalitolerant Nitrospira species

Nitrite-oxidizing enrichment cultures were established by inoculation of 40 ml sterile, mineral nitrite medium containing 0.5 mM filter-sterilized NaNO_2_ with approx. 0.1 g of fresh sediment or 1 ml of lake water. Enrichment cultures were incubated without agitation in 100 ml glass bottles in the dark at 28°C. Freshly prepared medium had a pH of 10.2 that decreased to 9 during approximately two months of cultivation. Actively nitrite-oxidizing enrichment cultures were further purified and propagated by 1:100 dilution in fresh nitrite medium. The nitrite and nitrate concentration in the medium were regularly checked by using nitrite/nitrate test stripes (Macherey-Nagel), and nitrite was repeatedly replenished when completely consumed. Precise chemical measurements [2] of the nitrite and nitrate concentrations were regularly performed during the early stages of the *Nitrospira* enrichment to confirm that nitrite was stoichiometrically oxidized to nitrate.

*Fluorescence* in situ *hybridization (FISH) and microscopy*

The abundance of *Nitrospira* in the enrichment cultures was regularly monitored by FISH with 16S rRNA gene-targeted probes that were doubly labeled with the fluorochromes Cy3, Cy5, or FLUOS. Probes targeting most bacteria (EUB338 probe mix at a formamide (FA) concentration of 35%; [3, 4], the phylum Nitrospirae (probe Ntspa712 with competitor at a FA concentration of 35%; [5]) and most lineage II plus some lineage IV species of the genus *Nitrospira* (probe Ntspa1151 at a FA concentration of 35%; [6]), the genus *Nitrobacter* (probe Nit3 with competitor at a FA concentration of 35%; [7]), the genus *Nitrotoga* (probe Ntoga122 with two competitors at a FA concentration of 40%; [8]) and probe NON338 [9] as a control for nonspecific probe binding were applied. To specifically detect lineage II *Nitrospira* only, probe Ntspa1151 was used in equimolar concentrations at a FA concentration of 35% with two newly designed, unlabeled competitor oligonucleotides targeting lineage IV *Nitrospira* (c1Ntspa1151: 5‘-TTA TCC TGG GCA GTC TCT CC-3’ and c2Ntspa 1151: 5‘-TTA TCC TGG GCA GTC TCT TC-3’). FISH was combined with nonspecific fluorescent labeling of all cells by 4’,6’-diamidino-2-phenylindole (DAPI). For FISH, aliquots of the enrichment cultures were formaldehyde-fixed and FISH was performed according to standard protocols [10]. Fluorescence micrographs of probe-stained organisms were acquired with an inverted Leica TCS SP8X confocal laser scanning microscope that was equipped with a 405 nm UV diode, a Leica supercontinuum white light laser, two photomultiplier (PMT) detectors, three hybrid (HyD) detectors, and the Leica Application Suite AF 3.2.1.9702.

*Physiological tests with* Nitrospira *enrichment cultures*

To determine the optimal pH conditions for growth, three of the initial *Nitrospira* enrichments (EN_A, EN_B, EN_C) were cultured for five weeks with repeated nitrite additions in the mineral medium as described above. The pH was adjusted to 7.6 or 9.0 by titration with 1M HCl, monitored throughout the duration of the incubation and adjusted when necessary. All incubations were performed in duplicates. Enrichments which had been cultured at around pH 9 were incubated for an additional period of 25 days, during which the pH was raised sequentially every eight or nine days to 10, 10.5, and finally to 11 by titration with 1 M NaOH. The optimal nitrite concentration for cultivation was determined using the enrichment culture of “Ca. N. alkalitolerans”. The culture was incubated in the presence of 0.15, 0.3, 0.7, and 1 mM NaNO_2_ for 30 days. In all physiological experiments, formaldehyde-fixed culture aliquots were incubated as a negative control under the same conditions. The nitrite and nitrate concentrations were measured according to Miranda *et al.* [2].

*Metagenome sequencing,* Nitrospira *genome assembly, and genome annotation*

An Illumina sequencing library was prepared from the DNA sample of the “*Ca.* N. alkalitolerans” enrichment using the ruSeq DNA PCR-free sample preparation (Illumina) following the manufacturer’s recommendations and paired-end sequenced (2×300 bp) twice on a MiSeq using a MiSeq Reagent kit v3 (Illumina) following the manufacturer’s recommendations. Base calling was carried out using MiSeq control software v.2.5. Illumina read quality and adaptor trimming (trim limit: 0.01, no ambiguous bases, min length: 55 bp), de novo assembly (word size: 21, bubble size: 186, min length: 500 bp), and read mapping (default settings except length fraction: 0.95 and similarity fraction: 0.95) were performed in CLC Genomics Workbench v. 8.5.1. The Illumina de novo assembly was checked for contamination and completeness using the mmgenome workflow (http://madsalbertsen.github.io/mmgenome/).

The most abundant scaffolds in the assembly were identified as Nitrospirae and binned into a provisional metagenome assembled genome (MAG) using mmgenome as described above. This MAG was further separated into three sub-bins based on coverage. Each of these sub-bins underwent iterative reassembly using MAGspinner (<https://github.com/hexaquo/MAGspinner>) which is a recently updated and automated version (used in [11]) of the manual method used in [12–14]. MAGspinner calculates null models for tetranucleotide composition and coverage for a MAG, rejects scaffolds that are outside the model and then maps (BBMap v. 36.32) raw reads to scaffolds for reassembly in Spades v. 3.10.1 [15], using the scaffolds that fit the null model as “trusted contigs”. The reassembly was repeated (in blocks of ten rounds) until genome statistics became self-consistent (based on output from CheckM v. 1.0.7 “checkm qa --tab_table -o 2”, [16]). Self-consistency was established by calculating the correlation between all checkM-calculated statistics and the number of rounds over the previous ten rounds of reassembly. When no statistic was correlated with round number (p>0.05 for all statistics), the iterative procedure ceased. Of the three *Nitrospirae* MAG sub-bins, only the most abundant remained consistently high in coverage throughout the binning procedure. The coverage of the two less abundant sub-bins “drifted” upwards over the reassembly procedure, indicating that the organisms represented by them were closely related to the organism represented by the most abundant sub-bin and that these sub-bins could not be delineated confidently. The novelty of original and reassembled sub-bins were assessed against published *Nitrospirae* using genomic average nucleotide identity (gANI) calculated with gANI-MiSI [17] and average amino acid identity (AAI) as calculated using bidirectional best blastp hits aligned over at least 70% of the length of both genes. The % identity was weighted by query gene length. The calculations were repeated using each genome as query and target and the average of both calculations was reported.

The more abundant sub-bin was accepted as the final “*Ca.* N. alkalitolerans” genome bin and uploaded to the MicroScope platform [18] under the name “*Candidatus* Nitrospira alkalitolerans strain KS” for automatic gene prediction and annotation, which was amended manually where necessary for key genes of chemolithoautotrophic nitrite oxidation, energy metabolism, and adaptations to the haloalkaline environment. Genome statistics in Table S2 were collected with the integrated tools of the MicroScope platform.

*Identification and phylogenetic analysis of* “*Candidatus* Nitrospira alkalitolerans” *gene products putatively involved in haloalkalitolerance*

Homology searches for orthologous genes were performed using the OrthoFinder software with default settings [19]. Candidate genes for pH homeostasis and osmoregulation at alkaline conditions were searched manually by browsing the automated gene annotations and by using the BLAST tool of the MicroScope platform*.* Amino acid alignments of ATPase subunit *c* genes were constructed with Clustal Omega [20] and visualized with ESPript3 [21].

*Cloning, DNA extraction, amplicon sequencing, and phylogenetic analysis of 16S rRNA gene and* nxrB *amplicons*

PCR products of 16S rRNA gene fragments from *Nitrospira* enrichment cultures were generated using primers 8F (5` AGA GTT TGA TYM TGG CTC 3`) [22] and the *Nitrospira*-specific primer R1158 (5`CCC GTT MTC CTG GGC AGT 3`) [6] as described in Herbold *et al.* (2015). The 20 µl PCR reaction consisted of 1× Taq buffer (Fermentas), 0.2 mM dNTPmix (Fermentas), 2 mM MgCl2 (Fermentas), 0.025 U Taq DNA polymerase (Fermentas), 0.1 mg mL^-1^ bovine serum albumin, 1 μM of each of the forward and reverse primers, and 1 μL of enrichment culture that had been freeze-thawed three times. The primary melting step of the PCR assay was extended to 15 min, but otherwise cycling was conducted as performed by Herbold *et al.* [23]. The amplified, 550 bp-long *Nitrospira* 16S rRNA gene fragments were purified using QIAquick PCR Purification Kit (Qiagen) and cloned with the TOPO-TA cloning Kit (Fisher Scientific) according to the manufacturers’ instructions. Plasmids from successfully transformed *E. coli* cells were harvested with the Plasmid MiniPrep Kit (Quiagen) according to the manufacturer’s instructions. The cloned inserts were PCR-amplified using the M13 forward and reverse primers (included in the TOPO-TA cloning kit) according to the manufacturer’s instructions and were then Sanger sequenced (Microsynth Austria GmbH). Enrichments were screened for the presence of Nitrospina by PCR targeting the *nxrB* with primers F169/ Rb638 (5` TAC ATG TGG TGG AAC A 3`/ 5`CGR GAC TGA TCG ATC A 3`, [17]; reverse primer: personal communication from Sebastian Lücker). PCR and cycling procedures were as described in Herbold *et al.* [23] with the exception of an annealing temperature of 55°C.

For amplicon sequencing, DNA was extracted from environmental samples according to Angel *et al.* [24] and used as template for PCR with primer 8F/R1158 (see above) targeting the 16S rRNA gene, and primers Fa169/Ra638 (see above/5` CGG TTC TGG TCR ATC A 3`; [25]) targeting the *nxrB* of *Nitrospira*. PCR and amplicon library preparation for Illumina MiSeq sequencing were performed as described in Herbold *et al.* [23]. MiSeq sequencing was performed at Microsynth Austria GmbH. Paired end reads were processed and mapped to operational taxonomic unit (OTU) representatives as described in Herbold *et al.* [16] including chimera checks using the UPARSE pipeline [26, 27]. The 16S rRNA gene amplicons were 550 bp long and underwent quality filtering and trimming using a strategy that is based on the protocol for Illumina data in the Earth Microbiome Project (Version 5 2012, [28]. Resulting library sizes ranged between 1172 and 8165 obtained sequences for the 16S rRNA gene data set and between 2507 and 10613 obtained sequences for the *nxrB* data set.

The species level thresholds for OTU clustering were 97% sequence identity for the 16S rRNA gene amplicons and 95% sequence identity for *nxrB* amplicons (the latter according to Pester *et al. [25]*). Non-target sequences were identified by aligning representative sequences from each OTU to the SILVA Ref data set Nr 99, release 132 [29] or to the *nxrB* reference data set from Pester and colleagues [25] with the integrated aligner and manual curation in ARB [30] and excluded from further analysis.

A maximum likelihood tree was generated in RaxML [31] using 30 full-length 16S rRNA gene sequences of selected reference *Nitrospira* species that had been aligned with the integrated aligner in ARB [30]. *Leptospirillum ferrooxidans* (AJ237903), *Ca.* Magnetobacterium bavaricum (FP929063), *Thermodesulfovibrio yellowstonii* DSM 11347 (CP001147) and *Ca.* Methylomirabilis oxyfera (FP565575) were used as outgroup. A 50% conservation filter was applied, resulting in 1310 valid alignment positions. The gamma model of rate heterogeneity and the generalized time-reversible (GTR) substitution model were utilized and 100 rapid bootstrap inferences were executed. The representative 16S rRNA gene sequences from each *Nitrospira* OTU and the 16S rRNA gene sequences from the three most abundant clones, were aligned to the set of full-length sequences and added to the maximum likelihood tree with the Evolutionary Placement Algorithm (EPA) using the GTRGAMMA substitution and rate heterogeneity model on 745 alignment positions.

*Geographical maps and Statistical analyses*

Generation of geographical maps and statistical analyses and were performed using the software R [32] version 3.3.2. Maps were drawn based on the data available in the package ‘maps’ and subsequently modified with Inkscape (www.inkscape.org).

16S rRNA gene sequence libraries were normalized with the GMPR method [33] prior to further analysis. Estimates of *Nitrospira* 16S rRNA gene OTU richness and the inverse Simpson’s index as a proxy for community diversity were calculated with the functions ‘estimatR’ and ‘diversity’ of the R package vegan [34]. Environmental factors determined in this study were centered and/or scaled using the function ‘scale’ and checked for auto-correlation with the function ‘pairs’. Since the dissolved organic matter concentrations correlated with the concentrations of total dissolved nitrogen, nitrite and iron, organic matter concentrations were excluded from further analysis. *Nitrospira* communities were grouped by hierarchical clustering of a Bray-Curtis dissimilarity matrix using the Ward agglomeration method with the command ‘hclust’. Principal coordinate analysis (PCoA) of *Nitrospira* communities was performed using the ‘cmdscale’ command. Differences in environmental conditions determined in this study between lakes were assessed by Welch’s unequal variances t-Test on scaled and centered data. Significance of differences in nitrite utilization and nitrate production by alkalitolerant *Nitrospira* enrichments over time were determined by correlation analysis with the function ‘cor.test’ using the Pearson method. Significant correlations (p ≤ 0.05) between environmental factors determined in this study and *Nitrospira* OTU richness and diversity were determined with the command ‘cor.test’ using the Spearman method.

**Supplemental Results**

*Adaptations to low iron availability*

Bioavailable iron is scarce in marine and alkaline systems [35, 36], and organisms living at elevated pH conditions use various mechanisms for iron sequestration [37, 38]. Like other *Nitrospira*, “*Ca.* N. alkalitolerans” must have a high demand for iron as key metabolic pathways of *Nitrospira*, such as nitrite oxidation, the electron transport chain, and CO_2_ fixation, depend on enzymes with iron-sulfur clusters or heme as cofactors [39]. Accordingly, the genome of “*Ca.* N. alkalitolerans” contains numerous genes for iron uptake and storage. Among them are six genes of putative TonB-dependent iron siderophore receptors, four gene copies of the energy-transducing inner membrane TonB*/*ExbB*/*ExbD complex, and several genes coding for bacterioferritin (Table S3). One of the TonB-dependent iron siderophore receptors is most similar to a homolog of the marine *Methylocaldum marinum* (47.3% AA identity). Interestingly, the genome of “*Ca.* N. alkalitolerans” does not appear to encode any known pathway for the synthesis of siderophores. Thus, we assume that this nitrite oxidizer scavenges siderophores produced by other organisms while saving the costs of siderophore biosynthesis from fixed inorganic carbon similar to *Nitrosomonas europaea* [40].

*Adaptation to toxic arsenit*e

High concentrations of inorganic arsenic have been found in several soda lakes and pristine aquifers [41–44], and the inhabiting microorganisms must possess mechanisms to deal with the presence of this highly toxic metal. The saline-alkaline lakes of the national park “Neusiedler See - Seewinkel” might also contain elevated levels of arsenite, as this has been reported for wetlands in the same area [45]. In the genome of “*Ca.* N. alkalitolerans”, we identified the gene *arsB*, which codes for a PMF-dependent arsenite resistance efflux pump of the ACR3 family [46] (Fig. 5, Table S3). Since this type of arsenite efflux pump is not present in any other characterized NOB, and the gene of “*Ca.* N. alkalitolerans” exhibits a high similarity to a homologous gene in *Nitrincola nitratireducens* (66.5% AA identity) isolated from a haloalkaline lake, its presence in the genome may be a specific adaptation of “*Ca.* N. alkalitolerans” to its extreme habitat.

**References**

1. Koch H, Galushko A, Albertsen M, Schintlmeister A, Gruber-Dorninger C, Lucker S, et al. Growth of nitrite-oxidizing bacteria by aerobic hydrogen oxidation. *Science* 2014; **345**: 1052–1054.

2. Miranda KM, Espey MG, Wink DA. A rapid, simple spectrophotometric method for simultaneous detection of nitrate and nitrite. *Nitric Oxide* 2001; **5**: 62–71.

3. Amann RI, Binder BJ, Olson RJ, Chisholm SW, Devereux R, Stahl DA. Combination of 16S rRNA-targeted oligonucleotide probes with flow cytometry for analyzing mixed microbial populations. *Appl Environ Microbiol* 1990; **56**: 1919–1925.

4. Daims H, Brühl A, Amann R, Schleifer K-H, Wagner M. The domain-specific probe EUB338 is insufficient for the detection of all bacteria: Development and evaluation of a more comprehensive probe set. *Syst Appl Microbiol* 1999; **22**: 434–444.

5. Daims H, Nielsen JL, Nielsen PH, Schleifer K-H, Wagner M. In situ characterization of *Nitrospira*-like nitrite-oxidizing bacteria active in wastewater treatment plants. *Appl Environ Microbiol* 2001; **67**: 5273–5284.

6. Maixner F, Noguera DR, Anneser B, Stoecker K, Wegl G, Wagner M, et al. Nitrite concentration influences the population structure of *Nitrospira*-like bacteria. *Environ Microbiol* 2006; **8**: 1487–1495.

7. Wagner M, Rath G, Koops H-P, Flood J, Amann R. In situ analysis of nitrifying bacteria in sewage treatment plants. *Water Sci Technol* 1996; **34**: 237–244.

8. Lücker S, Schwarz J, Gruber-Dorninger C, Spieck E, Wagner M, Daims H. *Nitrotoga*-like bacteria are previously unrecognized key nitrite oxidizers in full-scale wastewater treatment plants. *ISME J* 2014.

9. Wallner G, Amann R, Beisker W. Optimizing fluorescent in situ hybridization with rRNA-targeted oligonucleotide probes for flow cytometric identification of microorganisms. *Cytometry* 1993; **14**: 136–143.

10. Daims H, Stoecker K, Wagner M. Fluorescence in situ hybridisation for the detection of prokaryotes. In: Osborn AM, Smith CJ (eds). *Molecular Microbial Ecology*. 2005. Taylor & Francis, Abington, U.K., pp 213–239.

11. Kitzinger K, Marchant HK, Bristow LA, Herbold CW, Padilla CC, Kidane AT, et al. Single cell analyses reveal contrasting life strategies of the two main nitrifiers in the ocean. *Nat Commun* 2020; **11**: 1–12.

12. Dyksma S, Bischof K, Fuchs BM, Hoffmann K, Meier D, Meyerdierks A, et al. Ubiquitous Gammaproteobacteria dominate dark carbon fixation in coastal sediments. *ISME J* 2016; **10**: 1939–1953.

13. Mußmann M, Pjevac P, Krüger K, Dyksma S. Genomic repertoire of the Woeseiaceae /JTB255, cosmopolitan and abundant core members of microbial communities in marine sediments. *ISME J* 2017; **11**: 1276–1281.

14. Pjevac P, Dyksma S, Goldhammer T, Mujakić I, Koblížek M, Mußmann M, et al. In situ abundance and carbon fixation activity of distinct anoxygenic phototrophs in the stratified seawater lake Rogoznica. *Environ Microbiol* 2019; **21**: 3896–3908.

15. Bankevich A, Nurk S, Antipov D, Gurevich AA, Dvorkin M, Kulikov AS, et al. SPAdes: A new genome assembly algorithm and its applications to single-cell sequencing. *J Comput Biol* 2012; **19**: 455–477.

16. Parks DH, Imelfort M, Skennerton CT, Hugenholtz P, Tyson GW. CheckM: assessing the quality of microbial genomes recovered from isolates, single cells, and metagenomes. *Genome Res* 2015; gr.186072.114.

17. Varghese NJ, Mukherjee S, Ivanova N, Konstantinidis KT, Mavrommatis K, Kyrpides NC, et al. Microbial species delineation using whole genome sequences. *Nucleic Acids Res* 2015; gkv657.

18. Vallenet D, Belda E, Calteau A, Cruveiller S, Engelen S, Lajus A, et al. MicroScope—an integrated microbial resource for the curation and comparative analysis of genomic and metabolic data. *Nucleic Acids Res* 2013; **41**: D636–D647.

19. Emms DM, Kelly S. OrthoFinder: solving fundamental biases in whole genome comparisons dramatically improves orthogroup inference accuracy. *Genome Biol* 2015; **16**: 157.

20. Sievers F, Wilm A, Dineen D, Gibson TJ, Karplus K, Li W, et al. Fast, scalable generation of high-quality protein multiple sequence alignments using Clustal Omega. *Mol Syst Biol* 2011; **7**: 539.

21. Robert X, Gouet P. Deciphering key features in protein structures with the new ENDscript server. *Nucleic Acids Res* 2014; **42**: W320–W324.

22. Juretschko S, Timmermann G, Schmid M, Schleifer K-H, Pommerening-Röser A, Koops H-P, et al. Combined molecular and conventional analyses of nitrifying bacterium diversity in activated sludge: *Nitrosococcus mobilis* and *Nitrospira*-like bacteria as dominant populations. *Appl Environ Microbiol* 1998; **64**: 3042–3051.

23. Herbold CW, Pelikan C, Kuzyk O, Hausmann B, Angel R, Berry D, et al. A flexible and economical barcoding approach for highly multiplexed amplicon sequencing of diverse target genes. *Front Microbiol* 2015; **6**.

24. Angel R, Claus P, Conrad R. Methanogenic archaea are globally ubiquitous in aerated soils and become active under wet anoxic conditions. *ISME J* 2012; **6**: 847.

25. Pester M, Maixner F, Berry D, Rattei T, Koch H, Lücker S, et al. *NxrB* encoding the beta subunit of nitrite oxidoreductase as functional and phylogenetic marker for nitrite-oxidizing *Nitrospira*. *Environ Microbiol* 2014; **16**: 3055–3071.

26. Edgar RC. Search and clustering orders of magnitude faster than BLAST. *Bioinformatics* 2010; **26**: 2460–2461.

27. Edgar RC. UPARSE: highly accurate OTU sequences from microbial amplicon reads. *Nat Methods* 2013; **10**: 996–998.

28. Gilbert JA, Jansson JK, Knight R. The Earth Microbiome project: successes and aspirations. *BMC Biol* 2014; **12**: 69.

29. Yilmaz P, Parfrey LW, Yarza P, Gerken J, Pruesse E, Quast C, et al. The SILVA and “All-species Living Tree Project (LTP)” taxonomic frameworks. *Nucleic Acids Res* 2014; **42**: D643–D648.

30. Ludwig W, Strunk O, Westram R, Richter L, Meier H, Yadhukumar, et al. ARB: a software environment for sequence data. *Nucl Acids Res* 2004; **32**: 1363–1371.

31. Stamatakis A. RAxML version 8: a tool for phylogenetic analysis and post-analysis of large phylogenies. *Bioinformatics* 2014; **30**: 1312–1313.

32. R Core Team. R: A language and environment for statistical computing. 2013.

33. Chen L, Reeve J, Zhang L, Huang S, Wang X, Chen J. GMPR: A robust normalization method for zero-inflated count data with application to microbiome sequencing data. *PeerJ* 2018; **6**.

34. Oksanen J, Blanchet, FG, Kindt R, Legendre P, Minchin PR, O’Hara RB, et al. Vegan: community ecology package. R package version 1.17-1. Available at: http://CRAN.R-project.org/package=vegan. Accessed 1 Jan 2011.

35. Drechsel H, Jung G. Peptide siderophores. *J Pept Sci Off Publ Eur Pept Soc* 1998; **4**: 147–181.

36. Bruland KW, Orians KJ, Cowen JP. Reactive trace metals in the stratified central North Pacific. *Geochim Cosmochim Acta* 1994; **58**: 3171–3182.

37. McMillan DGG, Velasquez I, Nunn BL, Goodlett DR, Hunter KA, Lamont I, et al. Acquisition of iron by alkaliphilic *Bacillus* species. *Appl Environ Microbiol* 2010; **76**: 6955–6961.

38. Serrano LOD, Camper AK, Richards AM. An overview of siderophores for iron acquisition in microorganisms living in the extreme. *BioMetals* 2016; **29**: 551–571.

39. Lücker S, Wagner M, Maixner F, Pelletier E, Koch H, Vacherie B, et al. A *Nitrospira* metagenome illuminates the physiology and evolution of globally important nitrite-oxidizing bacteria. *Proc Natl Acad Sci* 2010; **107**: 13479–13484.

40. Chain P, Lamerdin J, Larimer F, Regala W, Lao V, Land M, et al. Complete genome sequence of the ammonia-oxidizing bacterium and obligate chemolithoautotroph *Nitrosomonas europaea*. *J Bacteriol* 2003; **185**: 2759–2773.

41. Nordstrom DK. Worldwide occurrences of arsenic in ground water. *Science* 2002; **296**: 2143–2145.

42. Oremland RS, Kulp TR, Blum JS, Hoeft SE, Baesman S, Miller LG, et al. A microbial arsenic cycle in a salt-saturated, extreme environment. *Science* 2005; **308**: 1305–1308.

43. Lloyd JR, Oremland RS. Microbial transformations of arsenic in the environment: From soda lakes to aquifers. *Elements* 2006; **2**: 85–90.

44. Kulp TR, Han S, Saltikov CW, Lanoil BD, Zargar K, Oremland RS. Effects of imposed salinity gradients on dissimilatory arsenate reduction, sulfate reduction, and other microbial processes in sediments from two California soda lakes. *Appl Environ Microbiol* 2007; **73**: 5130–5137.

45. Michalek K, Lazowski W, Zechmeister T. Burgenländische-Feuchtgebiete-und-ihre-Bedeutung-im-Naturschutz.pdf. 2012. Naturschutzbund Burgenland, Vienna, Austria.

46. Rosen BP. The role of efflux in bacterial resistance to soft metals and metalloids. *Essays Biochem* 1999; **34**: 1–15.
