# Supplementary material for: Exploring the upper pH limits of nitrite oxidation: diversity, ecophysiology, and adaptive traits of haloalkalitolerant Nitrospira
Source: ISME J. 2020 Jul 24;14(12):2967–79. doi: 10.1038/s41396-020-0724-1 (PMC7784846; doi:10.1038/s41396-020-0724-1)
Supplement: Supplementary file 2 — Figure S1 [file 41396_2020_724_MOESM2_ESM.pdf]

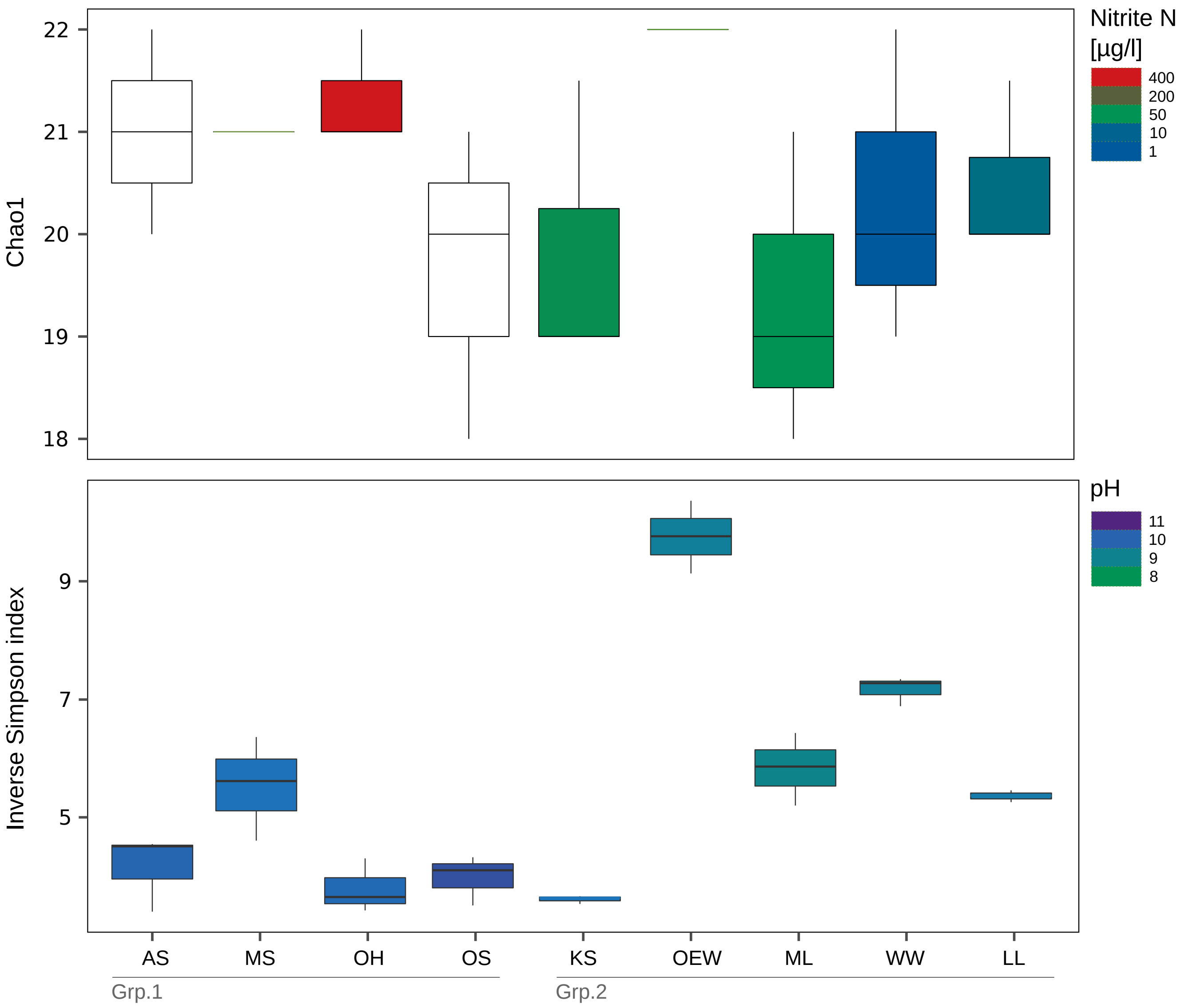

Figure S1 Estimations of alpha diversity (richness indicator Chao1 and inverse Simpson's index) for the Nitrospira OTUs from the analyzed saline-alkaline lakes. The Nitrospira OTUs were retrieved from the 16S rRNA gene amplicon dataset using a sequence identity threshold of 97%. Boxes are colored according to nitrite concentrations and pH values in lakes in the the upper and lower panel, respectively. Non-colored boxes denote missing data. Abbreviations for the lakes are the same as in main text Fig. 1; Grp.1, group 1 lakes; Grp.2, group 2 lakes (see also main text Fig. 1).
