# Supplementary material for: Exploring the upper pH limits of nitrite oxidation: diversity, ecophysiology, and adaptive traits of haloalkalitolerant Nitrospira
Source: ISME J. 2020 Jul 24;14(12):2967–79. doi: 10.1038/s41396-020-0724-1 (PMC7784846; doi:10.1038/s41396-020-0724-1)
Supplement: Supplementary file 3 — Figure S2 [file 41396_2020_724_MOESM3_ESM.pdf]

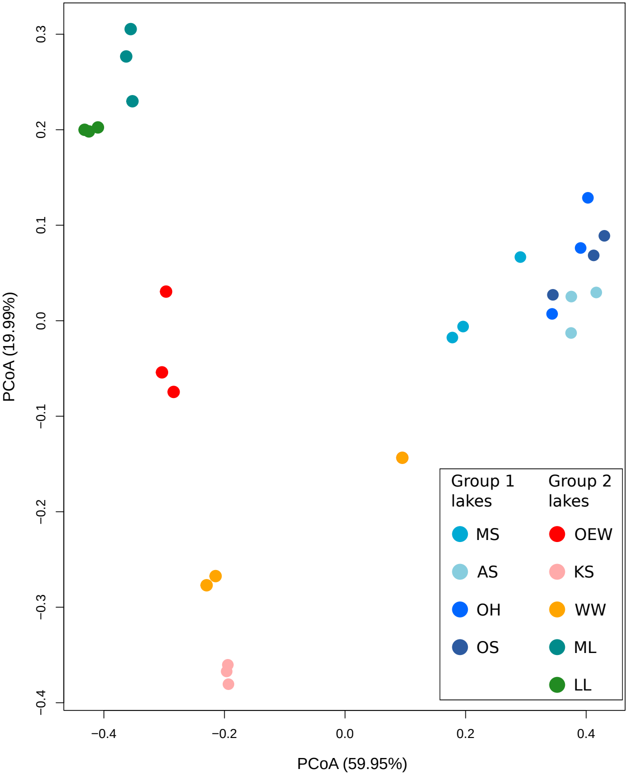

Figure S2 Biplot showing the first two axes of a principal coordinate analysis of the *Nitrospira* communities (OTUs) in nine saline-alkaline lakes grouped by hierarchical clustering of a Bray-Curtis dissimilarity matrix using the Ward agglomeration method. The axes cumulatively explain 79.94% of the observed variation. *Nitrospira* OTUs were retrieved from the 16S rRNA gene amplicon dataset using a sequence identity threshold of 97%. Lake identifiers and grouping of lakes are the same as in main text Fig. 1.
