# Supplementary material for: Exploring the upper pH limits of nitrite oxidation: diversity, ecophysiology, and adaptive traits of haloalkalitolerant Nitrospira
Source: ISME J. 2020 Jul 24;14(12):2967–79. doi: 10.1038/s41396-020-0724-1 (PMC7784846; doi:10.1038/s41396-020-0724-1)
Supplement: Supplementary file 4 — Figure S3 [file 41396_2020_724_MOESM4_ESM.pdf]

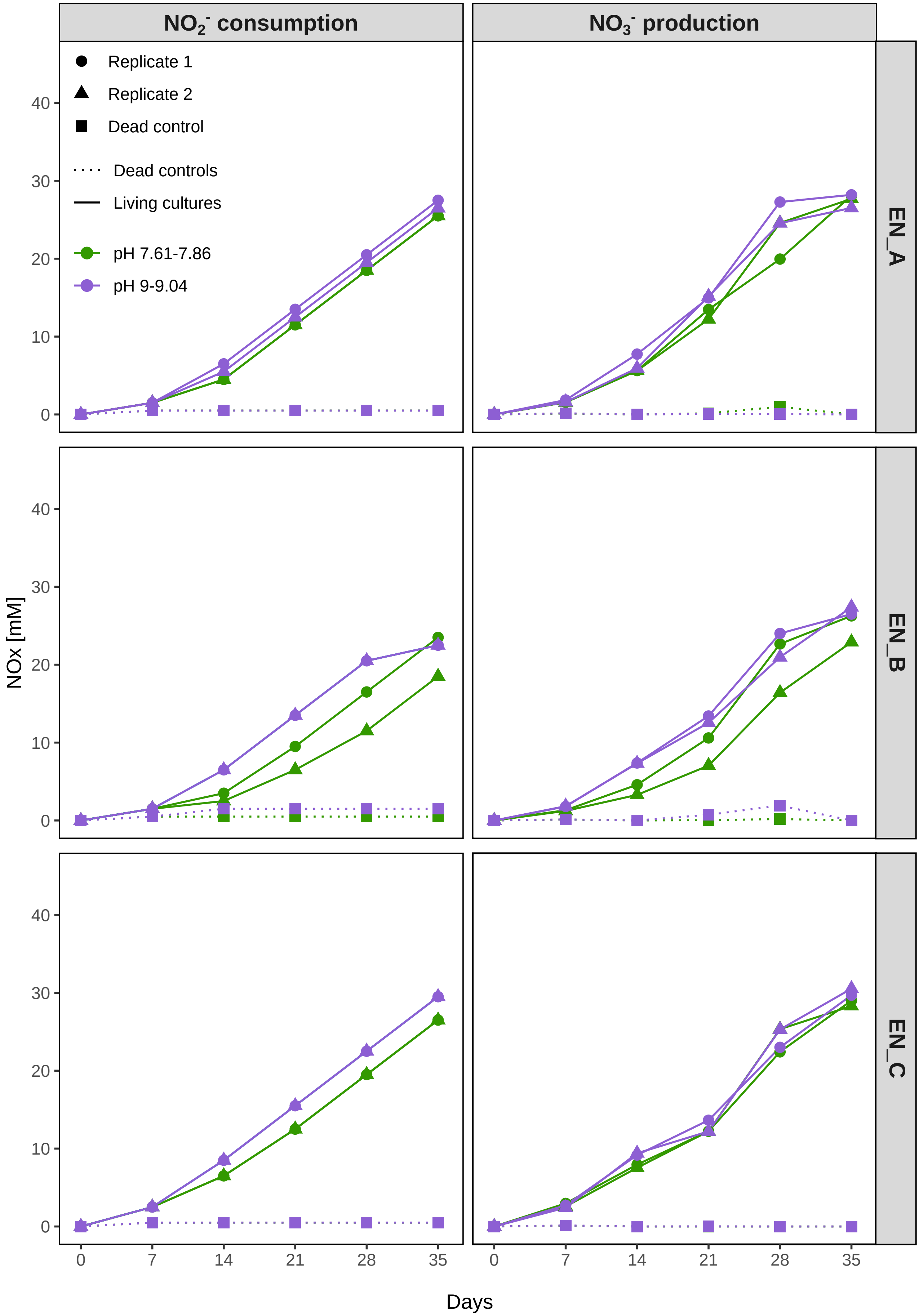


**Figure S3** Cumulative nitrite consumption and nitrate production, during 35 days by three alkalitolerant *Nitrospira* enrichment cultures from saline-alkaline lakes. The cultures were grown in mineral nitrite medium at pH 7.61-7.86 and 9-9.04. Data from two replicate incubations and one dead biomass control per pH treatment and enrichment are shown. Some symbols of replicate incubations or dead controls appear on top of each other. The pH was monitored and adjusted when necessary throughout the i ncubations (see table S3). EN_A, *Nitrospira* enrichment A; EN_B, *Nitrospira* enrichment B; EN_C, *Nitrospira* enrichment C.
