# Supplementary material for: Exploring the upper pH limits of nitrite oxidation: diversity, ecophysiology, and adaptive traits of haloalkalitolerant Nitrospira
Source: ISME J. 2020 Jul 24;14(12):2967–79. doi: 10.1038/s41396-020-0724-1 (PMC7784846; doi:10.1038/s41396-020-0724-1)
Supplement: Supplementary file 5 — Figure S4 [file 41396_2020_724_MOESM5_ESM.pdf]

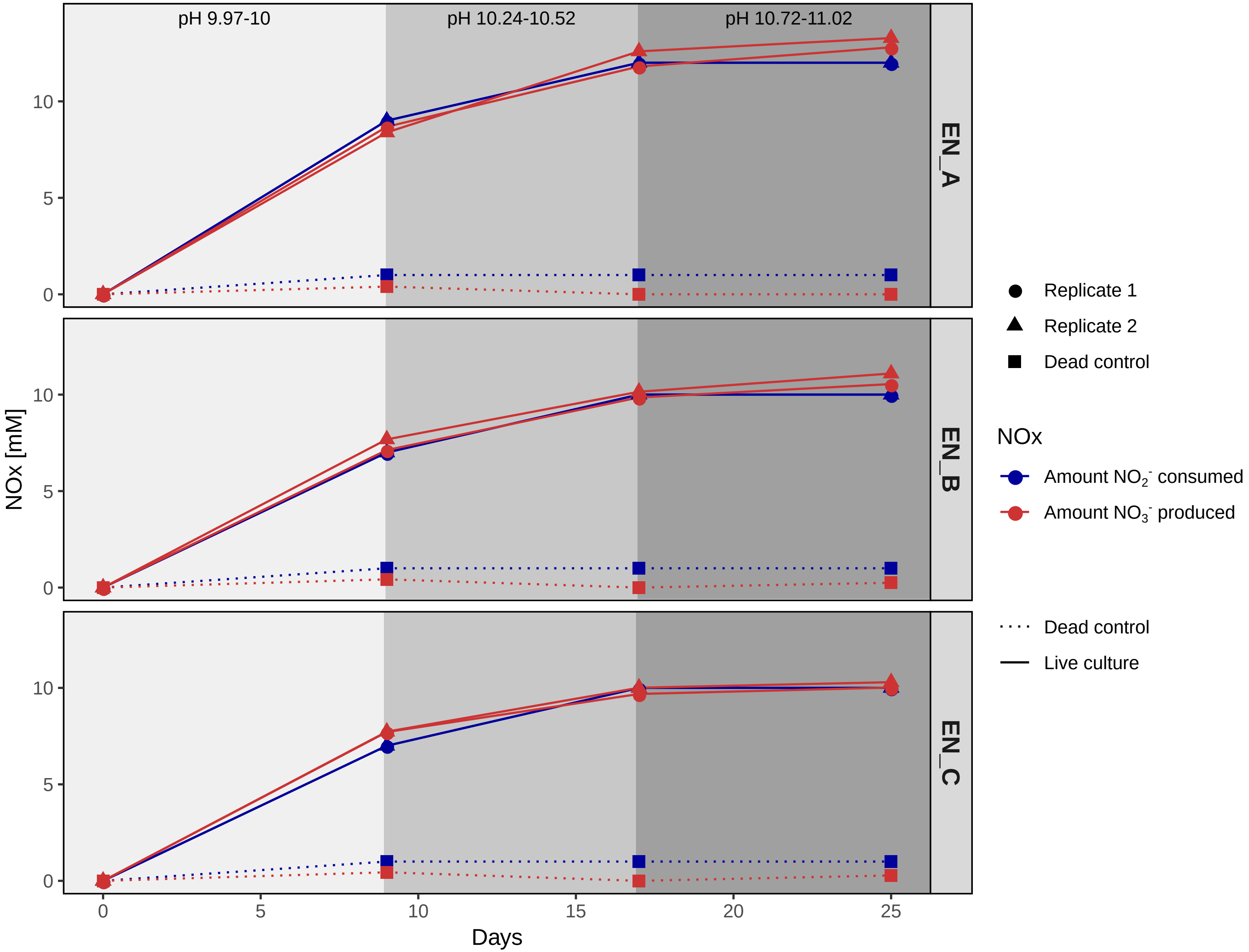

Figure S4 Cumulative nitrite consumption and nitrate production at elevated pH conditions (up to pH 11) by three alkalitolerant *Nitrospira* enrichment cultures from saline-alkaline lakes. The cultures were grown in mineral nitrite medium and the pH was sequentially raised from 9.97-10 to 10.24-10.52 and to 10.72-11.02. The pH was monitored and adjusted when necessary throughout the incubations (see table S3). Data from two replicate incubations and one dead biomass control per pH treatment are shown. Some symbols of replicate incubations appear on top of each other. EN-A, *Nitrospira* enrichment A; EN-B, *Nitrospira* enrichment B; EN-C, *Nitrospira* enrichment C.
