# Supplementary material for: Exploring the upper pH limits of nitrite oxidation: diversity, ecophysiology, and adaptive traits of haloalkalitolerant Nitrospira
Source: ISME J. 2020 Jul 24;14(12):2967–79. doi: 10.1038/s41396-020-0724-1 (PMC7784846; doi:10.1038/s41396-020-0724-1)
Supplement: Supplementary file 8 — Figure S7 [file 41396_2020_724_MOESM8_ESM.pdf]

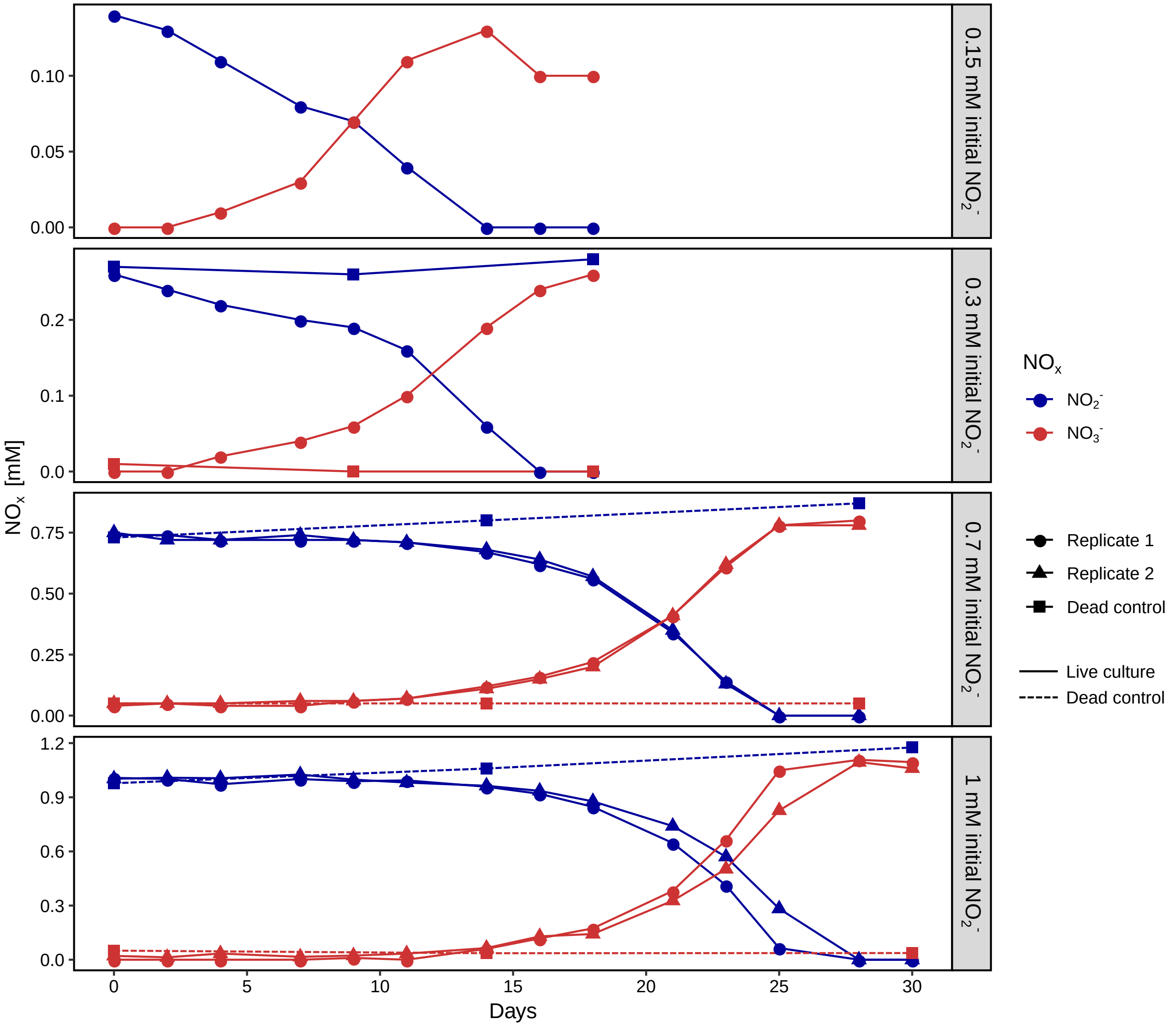


**Figure S7** Nitrite consumption and nitrate production of the "*Ca.* Nitrospira alkalitolerans" enrichment culture when cultured at pH 10.2 in mineral nitrite medium with 0.15, 0.3, 0.7, or 1 mM initial nitrite concentration. The pH was monitored and adjusted when necessary throughout the incubations. Treatments with 0.15 and 0.3 mM nitrite could not be replicated due to a lack of sufficient culture biomass.
