# Supplementary material for: Exploring the upper pH limits of nitrite oxidation: diversity, ecophysiology, and adaptive traits of haloalkalitolerant Nitrospira
Source: ISME J. 2020 Jul 24;14(12):2967–79. doi: 10.1038/s41396-020-0724-1 (PMC7784846; doi:10.1038/s41396-020-0724-1)
Supplement: Supplementary file 9 — Figure S8 [file 41396_2020_724_MOESM9_ESM.pdf]

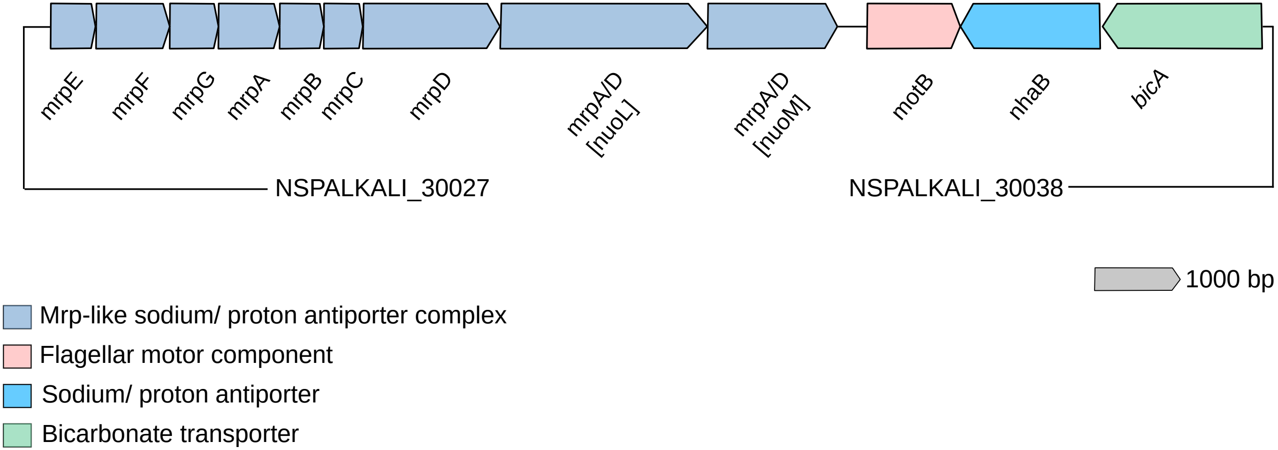

Figure S8 Schematic illustration of one of the genomic loci with a Mrp-like sodium/proton antiporter (*mrpA-E*) in "*Ca*. N. alkalitolerans". Two additional putative MrpA or MrpD subunits display sequence similarity to the complex I subunits NuoL and NuoM, respectively. Genes are drawn to scale.
