# Supplementary material for: Exploring the upper pH limits of nitrite oxidation: diversity, ecophysiology, and adaptive traits of haloalkalitolerant Nitrospira
Source: ISME J. 2020 Jul 24;14(12):2967–79. doi: 10.1038/s41396-020-0724-1 (PMC7784846; doi:10.1038/s41396-020-0724-1)
Supplement: Supplementary file 10 — Figure S9 [file 41396_2020_724_MOESM10_ESM.pdf]

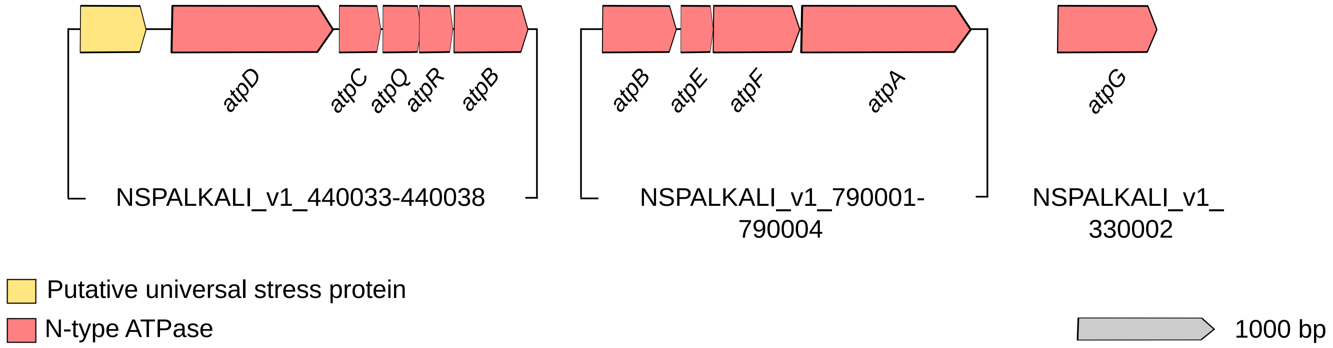

Figure S9 Schematic illustration of the genomic loci of the N-type ATPase with genes *atpA-G* of "*Ca.* N. alkalitolerans". Genes are drawn to scale.
