# Supplementary material for: Exploring the upper pH limits of nitrite oxidation: diversity, ecophysiology, and adaptive traits of haloalkalitolerant Nitrospira
Source: ISME J. 2020 Jul 24;14(12):2967–79. doi: 10.1038/s41396-020-0724-1 (PMC7784846; doi:10.1038/s41396-020-0724-1)
Supplement: Supplementary file 12 — Table S1 [file 41396_2020_724_MOESM12_ESM.pdf]

Table S1 pH monitoring and adjustments during the pH experiments with enrichment cultures A, B and C

Day	Treatment pH	pH measured	pH adjusted to
0	7.6	7.63 ± 0.02	-
3	7.6	7.86 ± 0.12	7.61 ± 0.02
5	7.6	7.84 ± 0.1	7.64 ± 0.04
8	7.6	7.78 ± 0.06	7.63 ± 0.01
11	7.6	7.82 ± 0.09	7.61 ± 0.02
15	7.6	7.75 ± 0.07	7.62 ± 0.03
17	7.6	7.78 ± 0.04	7.6 ± 0.02
22	7.6	7.82 ± 0.08	7.62 ± 0.01
27	7.6	7.79 ± 0.06	7.62 ± 0.01
31	7.6	7.76 ± 0.09	7.62 ± 0.02
35	7.6	7.78 ± 0.06	7.61 ± 0.03
0	9	9.03 ± 0.04	-
3	9	9.02 ± 0.06	-
8	9	9.03 ± 0.05	-
11	9	9 ± 0.06	-
17	9	9.02 ± 0.07	-
27	9	9.04 ± 0.06	-
35	9	9.02 ± 0.08	-
0	10	10 ± 0.02	-
2	10	9.97 ± 0.04	-
4	10	10 ± 0.03	-
6	10	9.99 ± 0.04	-
9	10	9.98 ± 0.02	-
9	10.5	10.52 ± 0.03	-
10	10.5	10.24 ± 0.07	10.51 ± 0.05
11	10.5	10.49 ± 0.05	-
14	10.5	10.36 ± 0.08	10.52 ± 0.02
17	10.5	10.51 ± 0.06	-
17	11	11.02 ± 0.04	-
18	11	10.72 ± 0.13	10.98 ± 0.03
19	11	10.97 ± 0.05	-
20	11	10.87 ± 0.09	11.02 ± 0.02
22	11	10.98 ± 0.03	-
25	11	10.98 ± 0.04	-

Values are averages (n=6) with standard deviation
