# Supplementary material for: Exploring the upper pH limits of nitrite oxidation: diversity, ecophysiology, and adaptive traits of haloalkalitolerant Nitrospira
Source: ISME J. 2020 Jul 24;14(12):2967–79. doi: 10.1038/s41396-020-0724-1 (PMC7784846; doi:10.1038/s41396-020-0724-1)
Supplement: Supplementary file 13 — Table S2 [file 41396_2020_724_MOESM13_ESM.pdf]

Table S2 *Nitrospira* MAG statistics

**Attribute**	***Ca.* Nitrospira alkalitolerans**	***Nitrospira* sub-bin1***	***Nitrospira* sub-bin2***
Size (bp)	4.894.714	957.413	658.454
Completeness (%)	95.83	21.93	0
Contamination (%)	4.83	0	0
DNA G + C (%)	51.4	51	51.5
DNA scaffolds	87	293	217
Total genes	5107	1142	799
RNA genes	35	3	90
rRNA genes	3	0	0
tRNA genes	47	8	8
Amino acids encoded by tRNA genes (unique codons)	20 (46)	6 (8)	5 (8)
nxrA genes (incl. Fragments)	3	4	3
nxrB genes (incl. Fragments)	1	*1*	*0*
Pairwise gANI value to “Ca. N. alkalitolerans” bin	100	97.1	95.2
Oneway gAAI value to “Ca. N. alkalitolerans” bin	99.9	94.3	81.1

*original sub-bin, not re-assembled
