# Supplementary material for: Exploring the upper pH limits of nitrite oxidation: diversity, ecophysiology, and adaptive traits of haloalkalitolerant Nitrospira
Source: ISME J. 2020 Jul 24;14(12):2967–79. doi: 10.1038/s41396-020-0724-1 (PMC7784846; doi:10.1038/s41396-020-0724-1)
Supplement: Supplementary file 14 — Table S3 [file 41396_2020_724_MOESM14_ESM.pdf]

Table S3

Selected proteins of “*Ca.* Nitrospira alkalitolerans” with predicted functions in metabolic pathways and in adaptation to high alkalinity and salinity

**Central Carbon Metabolism**			
**Glycogen formation/degradation**	**MaGe Identifier**	**Gene**	**Product**
	NSPALKALI_v1_40047	*glgX*	Glycogen debranching enzyme
	NSPALKALI_v1_60125	*glgX*	Glycogen debranching enzyme
	NSPALKALI_v1_170014	*glgX*	Putative Glycogen debranching enzyme
	NSPALKALI_v1_40023	*glgP*	Glycogen phosphorylase
	NSPALKALI_v1_140097	*glgP*	Glycogen phosphorylase
	NSPALKALI_v1_60127	*glgB*	1,4-alpha-glucan branching enzyme
	NSPALKALI_v1_60129	*glgC*	Glucose-1-phosphate adenylyltransferase
	NSPALKALI_v1_240060	*glgC*	Glucose-1-phosphate adenylyltransferase
	NSPALKALI_v1_60051	*glgE*	Alpha-1,4-glucan:maltose-1-phosphate maltosyltransferase
	NSPALKALI_v1_680007	*pgm*	Phosphoglucomutase
	NSPALKALI_v1_30082	*glk*	Glucokinase
	NSPALKALI_v1_120061	*pgi*	Glucosephosphate isomerase
	NSPALKALI_v1_40085	*malQ*	4-alpha-glucanotransferase
	NSPALKALI_v1_360024	*rfbF*	Glucose-1-phosphate cytidylyltransferase
	NSPALKALI_v1_80165		Amylo-alpha-1,6-glucosidase
**(r)TCA cycle**	**MaGe Identifier**	**Gene**	**Product**
	NSPALKALI_v1_20059	*aclB*	ATP-citrate lyase, beta subunit
	NSPALKALI_v1_20060	*aclA*	ATP citrate lyase, alpha subunit
	NSPALKALI_v1_200042	*forB*	2-oxoglutarate:ferredoxin oxidoreductase, beta subunit
	NSPALKALI_v1_200043	*forC*	2-oxoglutarate:ferredoxin oxidoreductase, gamma subunit
	NSPALKALI_v1_200044	*forE*	2-oxoglutarate:ferredoxin oxidoreductase, epsilon subunit
	NSPALKALI_v1_200045	*forD*	2-oxoglutarate:ferredoxin oxidoreductase, delta subunit
	NSPALKALI_v1_200046	*forA*	2-oxoglutarate:ferredoxin oxidoreductase, alpha subunit
	NSPALKALI_v1_20019	*porD*	Pyruvate:ferredoxin oxidoreductase, delta subunit
	NSPALKALI_v1_20018	*porC*	Pyruvate:ferredoxin oxidoreductase, gamma subunit
	NSPALKALI_v1_20017	*porB*	Pyruvate:ferredoxin oxidoreductase, beta subunit
	NSPALKALI_v1_20016	*porA*	Pyruvate:ferredoxin oxidoreductase, alpha subunit
	NSPALKALI_v1_20015		CO dehydrogenase or Pyruvate:ferredoxin oxidoreductase, delta subunit

	NSPALKALI_v1_510019	*sdhA/ nadB*	Succinate dehydrogenase/fumarate reductase, flavoprotein subunit or L-aspartate oxidase
	NSPALKALI_v1_20130	*sdhA/ nadB*	Succinate dehydrogenase/fumarate reductase, flavoprotein subunit or L-aspartate oxidase
	NSPALKALI_v1_200063	*sdhA/ nadB*	Succinate dehydrogenase/fumarate reductase, flavoprotein subunit or L-aspartate oxidase
	NSPALKALI_v1_510021	*sdhC*	Succinate dehydrogenase, subunit C (fragment)
	NSPALKALI_v1_510020	*sdhB*	Succinate dehydrogenase/ fumarate reductase iron-sulfur protein
	NSPALKALI_v1_510018	*gltA*	Citrate synthase
	NSPALKALI_v1_750005	*gltA*	Citrate synthase 2
	NSPALKALI_v1_170072	*sucA*	Pyruvate/2-oxoglutarate dehydrogenase complex,dehydrogenase (E1) component, eukaryotic type, alpha subunit
	NSPALKALI_v1_190028		Pyruvate/2-oxoglutarate dehydrogenase complex, dehydrogenase (E1) component, eukaryotic type, alpha subunit
	NSPALKALI_v1_170073	*sucB*	Pyruvate/2-oxoglutarate dehydrogenase complex,dehydrogenase (E1) component, eukaryotic type, beta subunit
	NSPALKALI_v1_20057	*icd*	Fragment of isocitrate dehydrogenase
	NSPALKALI_v1_40102	*icd*	Putative isocitrate dehydrogenase (NADP)
	NSPALKALI_v1_40103	*icd*	Isocitrate dehydrogenase (NAD+)
	NSPALKALI_v1_100028	*fumC*	Fumarate hydratase (fumarase C),aerobic Class II
	NSPALKALI_v1_580009	*mdh*	Malate dehydrogenase
	NSPALKALI_v1_200065	*sucC*	Succinyl-CoA ligase [ADP-forming], subunit alpha
	NSPALKALI_v1_200064	*sucD*	Succinyl-CoA ligase [ADP-forming] subunit beta
	NSPALKALI_v1_200061	*acnA*	aconitate hydratase 1
	NSPALKALI_v1_90082	*lpdA*	2-oxo-acid dehydrogenase complex, Dihydrolipoamide dehydrogenase (E3) (fragment)
**Carbonate uptake**	**MaGe Identifier**	**Gene**	**Product**
	NSPALKALI_v1_30038	*bicA*	Bicarbonate transporter BicA or sulfate permease
	NSPALKALI_v1_40075	*bicA*	Bicarbonate transporter BicA or sulfate permease
	NSPALKALI_v1_120088		Carbonic anhydase
	NSPALKALI_v1_550019		Carbonic anhydase
**Hydrogen Metabolism**			
**Hydrogen Metabolism**	**MaGe Identifier**	**Gene**	**Product**
	NSPALKALI_v1_490036		Putative (NiFe) hydrogenase, beta subunit (group 3b)
	NSPALKALI_v1_160002		Nickel-dependent hydrogenase, large subunit (group 3b)
	NSPALKALI_v1_500001		Hydrogenase transcriptional regulatory protein HoxA
	NSPALKALI_v1_500003		Ni,Fe-hydrogenase, small subunit (group 2a)
	NSPALKALI_v1_500004		Ni,Fe-hydrogenase, large subunit (group 2a)
	NSPALKALI_v1_500007		Hydrogenase maturation protein (fragment)
	NSPALKALI_v1_500014	*hypF*	Hydrogenase maturation protein
	NSPALKALI_v1_500015	*hypC*	Hydrogenase assembly chaperone protein

	NSPALKALI_v1_500017	*hypD*	hydrogenase expression/ formation protein
	NSPALKALI_v1_500018	*hypE*	hydrogenase expression/ formation protein
	NSPALKALI_v1_500020	*hypA*	putative hydrogenase nickel incorporation protein
	NSPALKALI_v1_500021	*hypB*	hydrogenase accessory protein
**Nitrite Metabolism**			
**Nitrite oxidation**	**MaGe Identifier**	**Gene**	**Product**
	NSPALKALI_v1_140038	*nxrB*	Nitrite oxidoreductase beta subunit
	NSPALKALI_v1_140037	*nxrA*	Nitrite oxidoreductase alpha subunit
	NSPALKALI_v1_530013	*nxrA*	Nitrite oxidoreductase alpha subunit
	NSPALKALI_v1_530020	*nxrA*	Nitrite oxidoreductase alpha subunit (fragment)
	NSPALKALI_v1_150108	*nxrC*	Putative nitrite oxidoreductase, gamma subunit
	NSPALKALI_v1_70126	*nxrC*	Nitrite oxidoreductase gamma subunit
	NSPALKALI_v1_70134	*nxrC*	Nitrite oxidoreductase gamma subunit
**Respiratory Chain**			
**Complex I**	**MaGe Identifier**	**Gene**	**Product**
	NSPALKALI_v1_400013	*nuoA*	NADH-quinone oxidoreductase subunit A
	NSPALKALI_v1_400014	*nuoB*	NADH-quinone oxidoreductase subunit B
	NSPALKALI_v1_400015	*nuoC/D*	NADH-quinone oxidoreductase subunit C/D
	NSPALKALI_v1_400016	*nuoE*	NADH-quinone oxidoreductase subunit E
	NSPALKALI_v1_400017	*nuoF*	NADH-quinone oxidoreductase, subunit F
	NSPALKALI_v1_400018	*nuoG*	NADH-quinone oxidoreductase, subunitG
	NSPALKALI_v1_400019	*nuoH*	NADH-quinone oxidoreductase subunit H
	NSPALKALI_v1_400020	*nuoI*	NADH-quinone oxidoreductase subunit I
	NSPALKALI_v1_400021	*nuoJ*	NADH-quinone oxidoreductase, membrane subunit J
	NSPALKALI_v1_400022	*nuoK*	NADH-quinone oxidoreductase subunit K
	NSPALKALI_v1_400023	*nuoL*	NADH-quinone oxidoreductase, membrane subunit L
	NSPALKALI_v1_400024	*nuoM*	NADH-quinone oxidoreductase subunit M
	NSPALKALI_v1_400025	*nuoN*	NADH-quinone oxidoreductase subunit N2
	NSPALKALI_v1_250014	*nuoA*	NADH-quinone oxidoreductase subunit A
	NSPALKALI_v1_250015	*nuoB*	NADH-quinone oxidoreductase subunit B
	NSPALKALI_v1_250016	*nuoC*	NADH-quinone oxidoreductase subunit C
	NSPALKALI_v1_250017	*nuoD*	NADH-quinone oxidoreductase subunit D
	NSPALKALI_v1_250018	*nuoG*	NADH-quinone oxidoreductase, subunitG
	NSPALKALI_v1_250019	*nuoI*	NADH-quinone oxidoreductase subunit I

	NSPALKALI_v1_250020	*nuoJ*	NADH-quinone oxidoreductase, membrane subunit J
	NSPALKALI_v1_250021	*nuoK*	NADH-quinone oxidoreductase subunit K
	NSPALKALI_v1_250022	*nuoL*	NADH-quinone oxidoreductase, membrane subunit L
	NSPALKALI_v1_250023	*nuoM*	NADH-quinone oxidoreductase, membrane subunit M
	NSPALKALI_v1_250024	*nuoM*	NADH-quinone oxidoreductase, membrane subunit M
	NSPALKALI_v1_250025	*nuoN*	NADH-quinone oxidoreductase subunit N
	NSPALKALI_v1_580010	*nuoF*	NADH-quinone oxidoreductase subunit F 2
**Complex II**	**MaGe Identifier**	**Gene**	**Product**
	NSPALKALI_v1_510019	*sdhA/ nadB*	Succinate dehydrogenase/fumarate reductase, flavoprotein subunit or L-aspartate oxidase
	NSPALKALI_v1_510020	*sdhB*	Succinate dehydrogenase/ fumarate reductase iron-sulfur protein
	NSPALKALI_v1_510021	*sdhC*	Succinate dehydrogenase, subunit C (fragment)
	NSPALKALI_v1_20130	*sdhA/ nadB*	Succinate dehydrogenase/fumarate reductase, flavoprotein subunit or L-aspartate oxidase
	NSPALKALI_v1_200063	*sdhA/ nadB*	Succinate dehydrogenase/fumarate reductase, flavoprotein subunit or L-aspartate oxidase
**Complex III**	**MaGe Identifier**	**Gene**	**Product**
	NSPALKALI_v1_150103		Putative Quinol-cytochrome c reductase, cytochrome b subunit
	NSPALKALI_v1_150102		Putative Quinol-cytochrome c reductase, iron-sulfur subunit, modulated with PRC-barrel (Modular protein)
	NSPALKALI_v1_370011		Menaquinol-cytochrome c reductase cytochrome b subunit
	NSPALKALI_v1_370010		Putative Quinol-cytochrome c reductase, iron-sulfur subunit (Rieske iron-sulfur protein)
**Complex IV**	**MaGe Identifier**	**Gene**	**Product**
	NSPALKALI_v1_150105		Putative Cytochrome bd-type quinol oxidase subunit 1
	NSPALKALI_v1_530011		Putative Cytochrome bd-type quinol oxidase, subunit 1
	NSPALKALI_v1_70108		Cytochrome bd ubiquinol oxidase subunit I
	NSPALKALI_v1_220083		Putative Cytochrome bd ubiquinol oxidase, subunit I
	NSPALKALI_v1_220084		Putative Cytochrome bd ubiquinol oxidase, subunit II
**Complex V**	**MaGe Identifier**	**Gene**	**Product**
	NSPALKALI_v1_10079	*atpH*	ATP synthase subunit delta
	NSPALKALI_v1_10080	*atpA*	F1 sector of membrane-bound ATP synthase, alpha subunit
	NSPALKALI_v1_10081	*atpD*	Membrane-bound ATP synthase , F1 sector, beta-subunit
	NSPALKALI_v1_10082	*atpC*	ATP synthase epsilon chain
	NSPALKALI_v1_790004	*atpA*	ATP synthase subunit alpha 2
	NSPALKALI_v1_20126	*atpI*	Putative ATP synthase F0, subunit I
	NSPALKALI_v1_20125	*atpB*	ATP synthase subunit a
	NSPALKALI_v1_20124	*atpE*	ATP synthase subunit c
	NSPALKALI_v1_20123	*atpF*	ATP synthase subunit b

	NSPALKALI_v1_120045	*atpG*	ATP synthase gamma chain
	NSPALKALI_v1_330007	*atpG*	ATP synthase gamma chain
	NSPALKALI_v1_10070	*yidC*	Membrane protein insertase YidC
**Iron uptake and storage**			
	**MaGe Identifier**	**Gene**	**Product**
	NSPALKALI_v1_170059		Bacterioferritin (modular protein)
	NSPALKALI_v1_60072		Bacterioferritin, iron storage and detoxification protein
	NSPALKALI_v1_80012	*fecE*	Iron-dicitrate transporter
	NSPALKALI_v1_110032	*fur*	Ferric uptake regulation protein
	NSPALKALI_v1_10210	*fur*	Ferric uptake regulation protein
	NSPALKALI_v1_58005		putative Bacterioferritin-associated ferredoxin
	NSPALKALI_v1_460022		putative TonB-dependent receptor
	NSPALKALI_v1_250054		putative TonB-dependent receptor
	NSPALKALI_v1_570021		putative TonB-dependent receptor
	NSPALKALI_v1_110038		putative TonB-dependent receptor
	NSPALKALI_v1_340011		putative TonB-dependent receptor
	NSPALKALI_v1_400004		putative Protein tonB2
	NSPALKALI_v1_400007	*exbB*	Biopolymer transport protein
	NSPALKALI_v1_570025	*exbD*	Biopolymer transport protein
	NSPALKALI_v1_570024	*exbD*	Biopolymer transport protein
	NSPALKALI_v1_140114	*exbD*	Biopolymer transport protein
**Flagellum biosynthesis**			
**Flagellum assembly**	**MaGe Identifier**	**Gene**	**Product**
	NSPALKALI_v1_190007	*motA*	Flagellar motor protein MotA
	NSPALKALI_v1_190008	*motB*	Flagellar motor protein MotB
	NSPALKALI_v1_140115		MotA/TolQ/ExbB proton channel family protein
	NSPALKALI_v1_30036	*ompA/ motB*	putative OmpA/MotB
**K^+ transport**			
**K^+ uptake**	**MaGe Identifier**	**Gene**	**Product**
	NSPALKALI_v1_150036		putative potassium channel NAD-binding component
	NSPALKALI_v1_270047	*trkC*	Trk system potassium uptake protein C
	NSPALKALI_v1_170093	*trkB*	Trk system potassium uptake protein B
	NSPALKALI_v1_100131	*trkB*	Trk system potassium uptake protein B
**K^+ efflux**	**MaGe Identifier**	**Gene**	**Product**

	NSPALKALI_v1_160038	*kefB*	putative K(+) efflux antiporter KefB
	NSPALKALI_v1_30114	*kefB*	Putative Glutathione-regulated potassium-efflux system
	NSPALKALI_v1_30115	*kefB*	Putative Glutathione-regulated potassium-efflux system
**Na^+ transport**			
**Sodium import**	**MaGe Identifier**	**Gene**	**Product**
	NSPALKALI_v1_50050		Sodium/solute symporter, putative (fragment)
	NSPALKALI_v1_140059		Sodium:dicarboxylate symporter
	NSPALKALI_v1_150072		Uncharacterized sodium-dependent transporter YhdH
	NSPALKALI_v1_110138	*agcS*	Sodium/alanine symporter
	NSPALKALI_v1_30038	*bicA*	Bicarbonate transporter BicA or sulfate permease
	NSPALKALI_v1_40075	*bicA*	Bicarbonate transporter BicA or sulfate permease
**Sodium/ cation exchange**	**MaGe Identifier**	**Gene**	**Product**
	NSPALKALI_v1_380014		Sodium/calcium exchanger
	NSPALKALI_v1_90107	*nhaA*	sodium:proton antiporter
	NSPALKALI_v1_30009	*nhaB*	fragment of sodium:proton antiporter (part 2)
	NSPALKALI_v1_30007	*nhaB*	fragment of sodium:proton antiporter (part 1)
	NSPALKALI_v1_30027	*mrpE*	putative Monovalent cation/H+ antiporter, subunit E
	NSPALKALI_v1_30028	*mrpF*	putative Monovalent cation/H+ antiporter, subunit F
	NSPALKALI_v1_30029	*mrpG*	putative Monovalent cation/H+ antiporter, subunit G
	NSPALKALI_v1_30030	*mrpA*	putative Monovalent cation/H+ antiporter, subunit A (Fragment, part 3)
	NSPALKALI_v1_30031	*mrpB*	putative Monovalent cation/H+ antiporter, subunit B
	NSPALKALI_v1_30032	*mrpC*	putative Monovalent cation/H+ antiporter, subunit C
	NSPALKALI_v1_30033	*mrpD*	putative Monovalent cation/H+ antiporter, subunit D
	NSPALKALI_v1_30034	*mrpA/D (nuoL)*	putative Monovalent cation/H+ antiporter, subunit A or D
	NSPALKALI_v1_30035	*mrpA/D (nuoM)*	putative Monovalent cation/H+ antiporter, subunit A or D
	NSPALKALI_v1_30037	*nhaB*	sodium:proton antiporter
	NSPALKALI_v1_40053	*mrpD*	putative Monovalent cation/H+ antiporter, subunit D
	NSPALKALI_v1_40054	*mrpC*	putative Monovalent cation/H+ antiporter, subunit C
	NSPALKALI_v1_40055	*mrpB*	putative Monovalent cation/H+ antiporter, subunit B
	NSPALKALI_v1_40056	*mrpA/B*	putative Monovalent cation/H+ antiporter, subunit A or B
	NSPALKALI_v1_40057	*mrpG/ phaG*	Monovalent cation/proton antiporter, MnhG/PhaG subunit
	NSPALKALI_v1_40058	*mrpF*	putative Monovalent cation/H+ antiporter, subunit F (Multiple resistance and pH regulation protein F)
	NSPALKALI_v1_40059	*mrpE*	putative Monovalent cation/H+ antiporter, subunit E
**Na^+ translocating complex I**			

**Alternative complex I**	**MaGe Identifier**	**Gene**	**Product**
	NSPALKALI_v1_330010	*nqrA*	Na(+)-translocating NADH-quinone reductase subunit A
	NSPALKALI_v1_330011	*nqrB*	Na(+)-translocating NADH-quinone reductase subunit B
	NSPALKALI_v1_330013	*nqrC*	Na(+)-translocating NADH-quinone reductase subunit C
	NSPALKALI_v1_330014	*nqrD*	Na(+)-translocating NADH-quinone reductase subunit D
	NSPALKALI_v1_330015	*nqrE*	Na(+)-translocating NADH-quinone reductase subunit E
	NSPALKALI_v1_330016	*nqrF*	Na(+)-translocating NADH-quinone reductase subunit F
**cbb3-type complex IV**			
**Alternative complex IV**	**MaGe Identifier**	**Gene**	**Product**
	NSPALKALI_v1_40069		Putative Cytochrome c oxidase (cbb3-type), fused subunits I, II, and III
**N-type ATPase**			
**Alternative Complex V**	**MaGe Identifier**	**Gene**	**Product**
	NSPALKALI_v1_440034	*atpD*	ATP synthase subunit beta
	NSPALKALI_v1_440035	*atpC*	H(+)-transporting ATP synthase, subunit epsilon
	NSPALKALI_v1_440036	*atpQ*	H(+)-transporting ATP synthase subunit q
	NSPALKALI_v1_440037	*atpR*	F1/F0 ATPase, Methanosarcina type, subunit r
	NSPALKALI_v1_440038	*atpB*	ATP synthase subunit a
	NSPALKALI_v1_790001	*atpB*	ATP synthase subunit a
	NSPALKALI_v1_790002	*atpE*	ATP synthase subunit c 2
	NSPALKALI_v1_790003	*atpF*	ATP synthase subunit b 2
	NSPALKALI_v1_790004	*atpA*	ATP synthase subunit alpha 2
	NSPALKALI_v1_330002	*atpG*	ATP synthase subunit gamma
**Osmoprotectants**			
**Glutamate synthesis**	**MaGe Identifier**	**Gene**	**Product**
	NSPALKALI_v1_70075	*gltB*	Glutamate synthase [NADPH], large subunit
	NSPALKALI_v1_70076	*gltD*	Glutamate synthase [NADPH], small chain
	NSPALKALI_v1_140012	*glt*	Putative glutamate synthase (Zinc finger CDGSH-type domain protein)
	NSPALKALI_v1_40009	*glt*	Putative glutamate synthase (Zinc finger CDGSH-type domain protein)
**Glycine betaine import**	**MaGe Identifier**	**Gene**	**Product**
	NSPALKALI_v1_140025	*opuD*	Glycine betaine transporter
	NSPALKALI_v1_20246	*opuCB*	ABC-type glycine betaine transport, periplasmic subunit
**Trehalose synthesis**	**MaGe Identifier**	**Gene**	**Product**
	NSPALKALI_v1_60049	*treS*	Trehalose synthase
**Other**			

Pathway	MaGe Identifier	Gene	Product
Membrane adaptation	NSPALKALI_v1_490030	*cls*	Cardiolipin synthase
Arsenical resistance	NSPALKALI_v1_370005	*arsB/ acr3*	Arsenite efflux pump ArsB, ACR3 family
